# Incidence, characteristics, and mortality of infective endocarditis in France in 2011

**DOI:** 10.1371/journal.pone.0223857

**Published:** 2019-10-25

**Authors:** S. Sunder, L. Grammatico-Guillon, A. Lemaignen, M. Lacasse, C. Gaborit, D. Boutoille, P. Tattevin, E. Denes, T. Guimard, M. Dupont, L. Fauchier, L. Bernard

**Affiliations:** 1 CH de Niort, Service des Maladies Infectieuses et Tropicale, Niort, France; 2 CHRU de Tours, Unité d’Épidémiologie des données cliniques, EpiDcliC, Tours, France; 3 Unité Inserm 1259, Université de tours, Tours, France; 4 CHRU de Tours, Service de Médecine Interne et Maladies Infectieuses, Tours, France; 5 CHU de Nantes, Service des Maladies Infectieuses et Tropicales, Nantes, France; 6 CHU de Rennes, Service des Maladies Infectieuses et Réanimation Médicale, Rennes, France; 7 CHU de Limoges, Service des Maladies Infectieuses et Tropicales, Limoges, France; 8 CH de La Roche sur Yon, Service des Maladies Infectieuses, La Roche sur Yon, France; 9 CH de Saint Malo, Service des Maladies Respiratoires et Infectieuses, Saint Malo, France; 10 Equipe d’accueil EA 1275, Université de Tours, Tours, France; 11 CHRU de Tours, Service de cardiologie, Tours, France; Rabin Medical Center, Beilinson Hospital, ISRAEL

## Abstract

**Objectives:**

We assessed the determinants of mortality in infective endocarditis (IE), using the national hospital discharge databases (HDD) in 2011.

**Methods:**

IE stays were extracted from the national HDD, with a definition based on IE-related diagnosis codes. This definition has been assessed according to Duke criteria by checking a sample of medical charts of IE giving a predictive positive value of 86.1% (95% confidence interval (CI): 82.7% - 89.5%). The impact of heart valve surgery on survival has been studied if performed during the initial stay, and over the year of follow-up. Risk factors of in-hospital mortality were identified using logistic regression model for the initial stay and Cox Time-dependent model for the 1-year mortality.

**Results:**

The analysis included 6,235 patients. The annual incidence of definite IEs was 63 cases/million residents. *Staphylococci* and *Streptococci* were the most common bacteria (44% and 45%, respectively). A valvular surgery was performed in 20% of cases, but substantial variations existed between hospitals. The in-hospital mortality was 21% (ranging 12% to 27% according to the region of patients), associated with age>70, chronic liver disease, renal failure, S. aureus, P. aeruginosa or candida infection and strokes whereas valvular surgery, a native valve IE or intraveinous drug use (right heart IE) were significantly protective for an initial death. The same factors were associated with the one-year mortality, except for valvular surgery which was associated with a 1.4-fold higher risk of death during the year post IE.

**Conclusion:**

We reported a high IE incidence rate. Valvular surgery was considerably less frequent in this study than in the previous published data (near 50%) whereas mortality was similar. Surgery was associated with higher survival if undergone within the initial stay. There were significant regional differences in frequency of surgery but it did not impact mortality.

## Introduction

In France in 2008, the annual incidence of IE (classified definite according to the Duke criteria [1]) was estimated at 33.8 cases per million inhabitants [2]. This incidence was calculated from an observational survey conducted in 2008 in seven French regions (covering over 22% of the French population). Cardiac surgery was performed for 45% of patients (223/497) and the overall mortality (in-hospital death) was 23%. These results are similar to other studies conducted in developed countries over the last decade [3–8].

Epidemiological data are generally issued from observational studies, more rarely from population based-studies and even more rarely nationwide studies. Recent studies based on hospital discharge databases (HDD) [6,7,9–13], aimed to describe the epidemiology of IE. In France, every hospital discharge (HD) must be registered in the French HDD, whatever the hospitalization sector. We recently conducted an epidemiological and economical study of IE on the regional HDD of a French region (between 2007 and 2009) [14]. The case definition of IE, using diagnosis codes (International Classification for Diseases–ICD10), was validated by a review of medical records of a sample of 200 patients and was robust (predictive positive value—PPV 86%). Some results differed substantially from the previous multicenter French population-based study [2], with a higher incidence of IE in the regional study estimation, a recourse of valvular surgery considerably less frequent, whereas mortality was similar. Facing the performance of HD algorithm of IE, extending these results at a national level by HDD analysis could refine our knowledge about IE epidemiology in France, and facilitate comparison with other countries. The objective of this study was to describe the epidemiology and clinical outcomes of IE in France in 2011, using national HDD codes.

## Material and methods

### Study design

A retrospective national cohort study of IE was performed using data collected from the national French HDD (NHDD) in 2011. Data for all patients hospitalized for IE were extracted from NHDD, using the HD algorithm defined below. We used the unique and encrypted patient number to link multiple hospitalizations to anonymized patient data, and thereby obtain the patient database. Follow-up started at the first hospital stay with IE diagnosis (admission day), with a minimal follow-up of 12 months (inclusion up to December 2011 and follow-up until December 2012). Patients were not recalled for this study but followed through their consecutive hospital stay discharges, regardless of the location of the admission in France, in- or outside their residency region.

### Case definition of IE

IE screening was based on the HD algorithm validated in our previous study [14]. This HD algorithm was developed by experts specialized in infectious diseases, cardiology and in medical information systems. Each stay with a primary or secondary diagnosis code (ICD-10) of IE, alone or in combination with either microbial or associated complication codes was retained (appendix). If the same patient had several hospital stays for IE during the one-year follow-up, we considered it as the same episode of IE. Outpatient visits were excluded from the analyses.

### Study variables

Variable used in the epidemiological analysis, evaluated as potential confounders, included patient variables (comorbidities) and hospital variables: age categorized into 10-year age groups, sex, comorbidities, causative pathogens, valve status (native valve, prosthetic valve), complications of IE, total length of stay, valve surgery and in-hospital mortality. The in-hospital mortality was defined as death during the initial stay or readmission. The case fatality rate was calculated using the number of stays with in-hospital death as numerator, and all IE patients as denominator. We calculated the annual incidence of IE, adjusted for PPV of the case definition and the frequency of definite IE according to Duke criteria.

Lost-to-follow-up in this HD cohort was defined as no further hospital readmission. For non survivors, date of death was recorded.

### Statistical analysis

The annual incidence of IE was calculated for the overall population, as well as by age and sex. The population data were derived from the 2011 census (*Insee*–French National Institute of Statistics and Economic Studies).

Results are presented as frequencies or as means and medians with range. The chi-squared test or Fisher test was used to compare categories. Logistic regression model was performed to analyze the factors associated with in-hospital mortality. All possible explanatory variables were first tested in a univariate model. The criterion for inclusion in the multivariate analysis was P<0.2 in the univariate analysis. We also performed a logistic regression model to assess the factors associated with valvular surgery during the initial hospitalization. In addition, we searched for factors associated with valvular surgery from IE diagnosis and initial treatment until one year follow-up, using a Cox model.

Survival analyses were performed analyzing occurrence of death at one year as the main outcome. First, Kaplan Meier curves described overall survival and all possible explanatory variables were first tested in an univariate model, including valvular surgery. The criterion for inclusion in the multivariate analysis was P<0.2 in the univariate analysis. Age and gender were always included. Cox proportional hazards models with time-dependent covariate (valvar surgery) were used to determine the effects of different confounding factors, as well as time period, on the risk of death. Hazard ratios (HR) and 95% confidence intervals (_95%_CI) were calculated. We checked proportionality of hazards and log-rank test. SAS software, version 9.2, for Microsoft Windows (SAS Institute, Cary, NC, USA) was used for statistical analysis.

## Results

### Validation of the case definition

The case definition was validated in 4 teaching hospitals and 3 general hospitals. We selected all the IE hospitalizations according to the algorithm definition, by extraction from the local HDD of these hospitals (n = 388). Data were extracted from medical records, and definite IE was defined using Duke criteria, as reported in the 2009 ESC guidelines for management of infective endocarditis [1]. The entire medical record of each patient was reviewed by one infectious disease specialist. Of the 388 cases of IE detected with the HD algorithm, 334 fulfilled Duke criteria for IE, which translates into a PPV of HD algorithm of 86.1% (95% confidence interval (CI): 82.7% - 89.5%), stable as compared with the regional study (PPV, 87%) [14]. Of the 334 IE, 251 were classified as definite IE, and 83 as possible IE. Hence, the PPV of HD algorithm for the diagnosis of definite IE was 75.1% (95% CI: 70.5% - 79.7%), whereas 64.7% for all HD cases.

### Epidemiology

In 2011, in France, 6,235 patients met the criteria for IE according to the case definition. Patients’ characteristics are summarized in Table 1. The annual incidence of definitive IE, using the estimated PPV of HD algorithm, was estimated at 63 cases/million inhabitants: 82 cases/million inhabitants for males, and 43 cases/million inhabitants for females (Sex ratio 1.9).

**Table 1:** Characteristics of IE patient, France 2011.

Characteristics	n = 6,235 (%)
Male gender	4,003 (*64*.*2*)
Age, mean, (median, 25%, 75%)	69 (72, 60, 81)
Predisposing diseases	
Chronic cardiac disease	1,701 (*27*.*3*)
Diabetes mellitus	1,479 (*23*.*7*)
Cancer	1,004 (*16*.*1*)
Chronic renal disease	1,251 (*20*.*1*)
Chronic respiratory disease	894 (*14*,*3*)
Intravenous drug use	125 (*2*.*0)*
Hemodialysis dependence	345 (*5*.*5*)
Valve status	
Native valve	4,488 (*72*.*0*)
Valve prosthesis	1,247 (*20*.*0*)
Unknown	500 (*8*.*0*)
Pacemaker	1,025(*16*.*4*)
Microorganism	
Monomicrobial	3,490 (*56*.*0*)
*Streptococci/Enterococci*	1,557(*44*.*6*)
*Staphylococci*	1,531 (*43*.*9*)
Gram-negative bacilli	317 (9.1)
*Candida* sp.	46 (1.3)
*Coxiella burnetii*	17 (0.5)
*Bartonella henselae*	3 (0,1)
Polymicrobial	513 (8.2)
Not documented	2,232 (*35*.*8*)
No microbiological code	1,994(*89*.*3*)
Code for non-specified pathogen	238 (*10*.*7*)
Complication	
Cardiogenic shock	511 (*8*.*2*)
Stroke	803 (*12*.*9*)
Ischemic stroke	586 (*73*.*0*)
Hemorrhagic stroke	217 (*27*.*0*)
Acute ischemia of lower limb	158 (*2*.*5*)
Vertebral osteomyelitis	292 (*4*.*7*)
Valvular surgery	1,576 (*25*.*3*)
During initial stay	635 (40.3)
During one year follow-up	941 (59.7)
Death	1,814 (29.1)
During initial IE hospital stay	1,283 (*70*.*7*)
Death during follow-up	531 (29.3)

**Fig 1:**
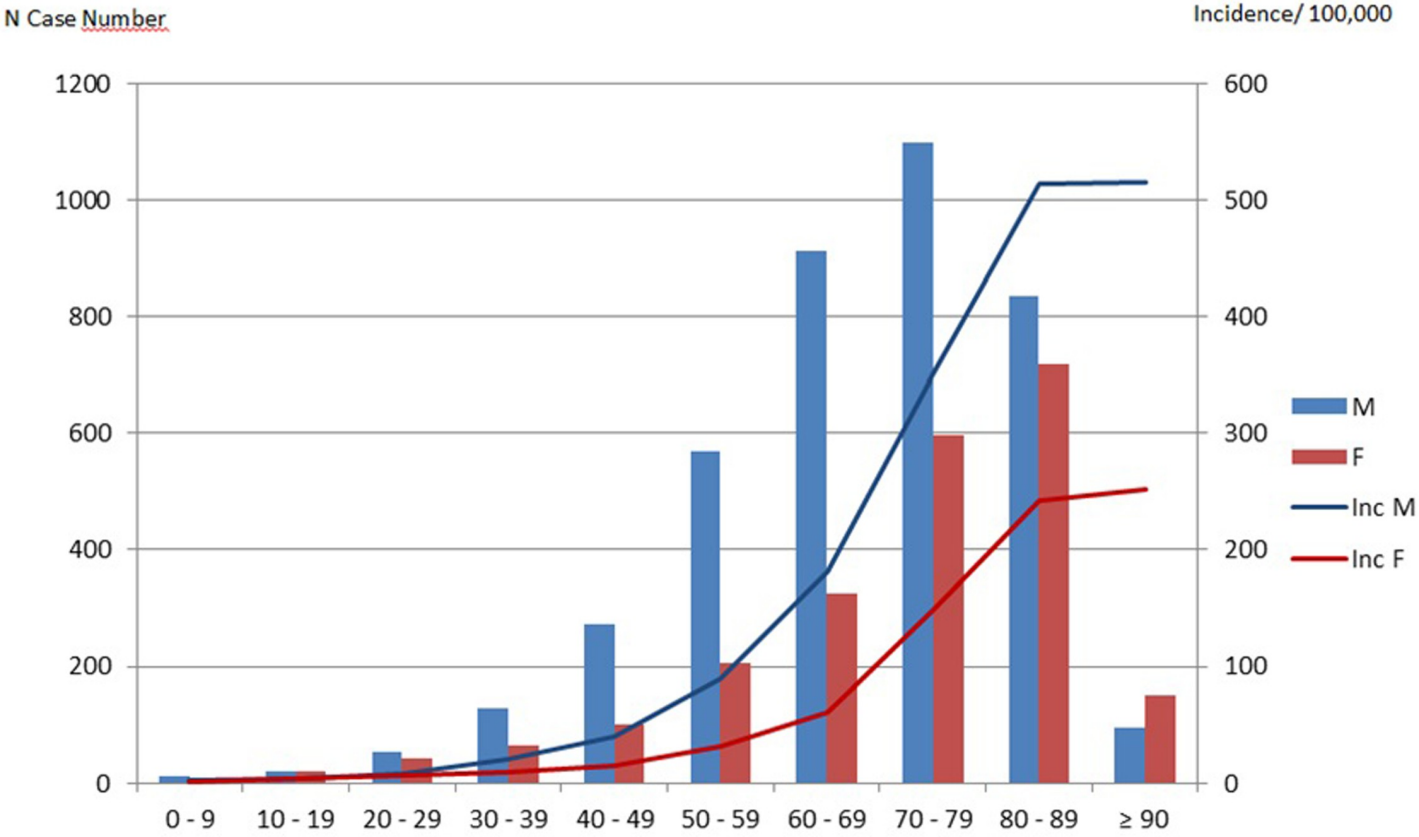
Distribution (histograms) and annual incidence (lines) of infective endocarditis, by sex and age, France 2011.

Mean age of IE patients was 69 years; IE incidence was higher in males whatever the age category and occurred in younger ages in males than females (mean age, 67.5 in males versus 71.6 years, p<0.001) (Fig 1). A substantial proportion of IE patients had comorbidities, the most frequent being chronic heart failure (27.3%), diabetes (23.7%), renal failure (20%) and cancer (16.1%). The mean length of the initial stay was 31.3 days.

Regarding valvular status, 1,247 patients (20%) were classified as prosthetic valve endocarditis (PVE). Baseline valvular status was unknown for 500 patients (8%), for whom it was impossible to settle if VP was the cause or the consequence of IE (i.e. patients who underwent valve replacement during the initial admission). Finally, 4,488 patients (72%) were classified as native valve (NV) IE. One thousand and twenty five patients (16.4%) had a pacemaker (PM).

### Microbiology

A microbiological code was reported in the records for 4,003 patients (64.2%): 238 patients (5.9%) had a code for non-specified bacterial infection, 513 (12.8%) had several microbiological codes, defining polymicrobial IE, and 3,490 patients (87.2%) had a single microbiological code (monomicrobial IE), with a predominance of *Streptococci/Enterococci* (44.6%) and *Staphylococci* (43.9%).

### Surgical management of IE

Valve surgery was performed in 1,576 patients (25.3%), including 635 (10.2% of the population) during the initial hospital stay, and 941 (15.1% of the IE population) between initial IE discharge and the one-year post IE hospital discharge. Factors associated with valvular surgery during the initial stay and one-year follow-up are presented in Table 2.

**Table 2:** Factors associated with valvular surgery.

	Surgery during initial stay adjusted Odds Ratio (95%CI), Multivariate logistic regression	Surgery during the global follow-up Adjusted Hazard ratio(95%CI),Cox model
Female	0.77 (0.61–0.97)	0.67 (0.58–0.79)
Age < 70 years	3.03 (2.43–3.77)	3.04 (2.63–3.51)
Predisposing diseases		
Cancer	0.51 (0.36–0.73)	0.52 (0.42–0.65)
Diabetes	NS	0.73 (0.61–0.87)
Chronic respiratory disease	NS	1.29 (1.05–1.57)
Chronic cardiac disease	NS	1.27 (1.09–1.49)
Obesity	NS	1.48 (1.19–1.84)
Valve status*		
Native valve	NS	NS
Prosthetic valve	NS	NS
Microbiology**		
*Staphylococcus aureus*	0.65 (0.50–0.85)	0.63 (0.52–0.75)
CNS	NS	1.33 (1.06–1.67)
Streptococci/Enterococci	0.70 (0.54–0.90)	1.38 (1.18–1.61)
*Escherichia coli*	NS	0.70 (0.50–0.99)
*Haemophilus spp*	NS	2.87 (1.18–7.01)
Complications		
Ischemic stroke	NS	1.30 (1.03–1.65)
Haemorragic stroke	0.36 (0.16–0.82)	0.67 (0.44–1.00)

CNS: coagulase-negative staphylococci.

### Survival analyses

The in-hospital mortality during the hospital stay for IE was 20.6%. The one-year mortality was 29.1%. The mortality of patients without any surgery procedure was 37.4%. The mortality during the initial IE hospital stay was associated with age>70, chronic liver disease, renal failure, *S*. *aureus*, *P*. *aeruginosa* or *Candida* infection and strokes whereas valvular surgery, a native valve IE and intravenous drug use were significantly protective for an initial death.

**Fig 2:**
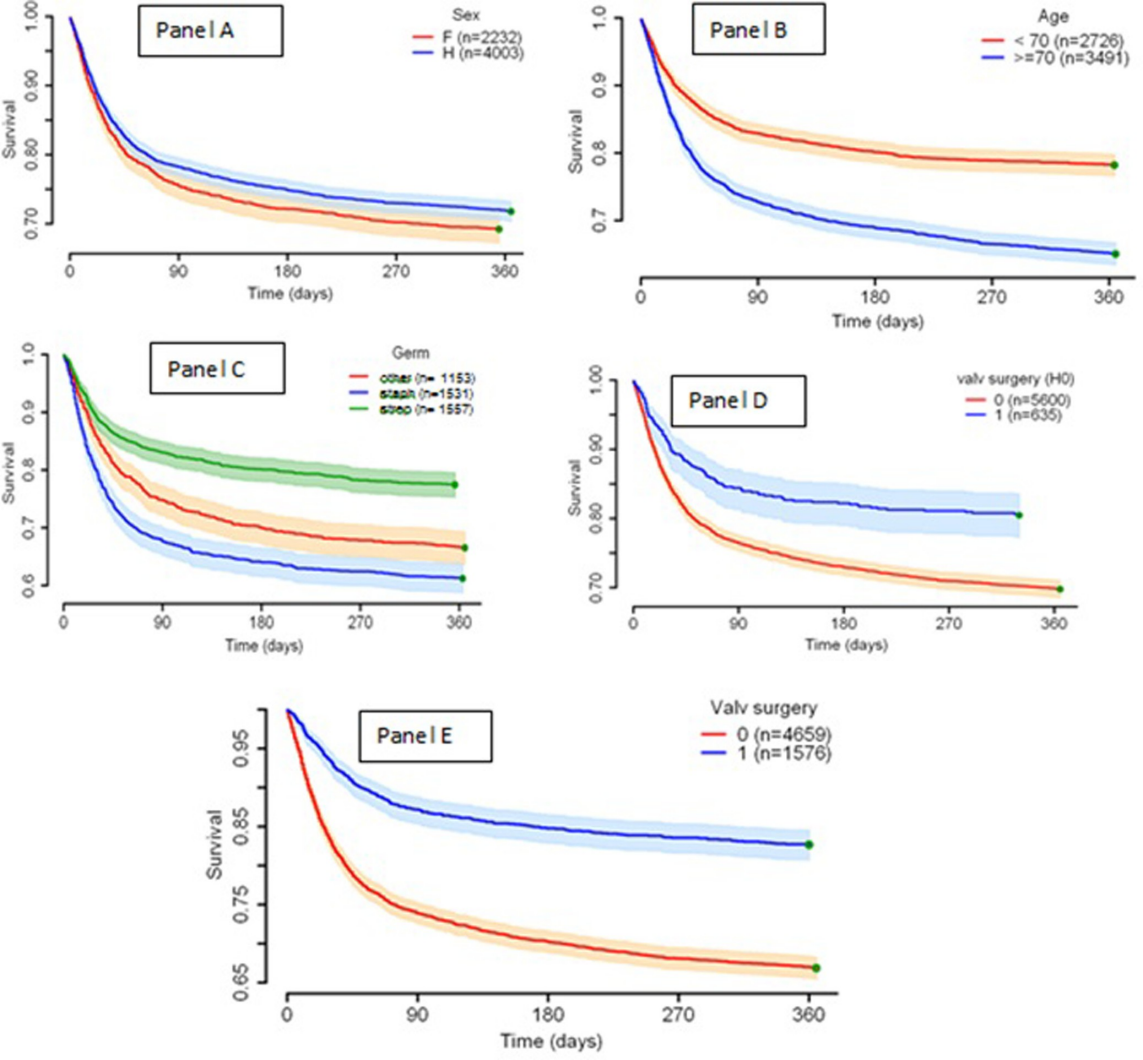
Kaplan Meier curves according to sex (panel A), age category (panel B), microorganism (panel C) or valvular surgery during the first IE hospitalization (H0, panel D), or within the one-year follow-up (panel E).

Kaplan Meier curves according to sex, age category, microbiology, or valvular surgery (during initial stay or global follow-up) are presented in Fig 2.

The factors predicting one-year mortality are presented in Table 3. There were the same risks during the initial IE diagnosis and treatment stay, except for valvular surgery which was associated with a 1.4-fold higher risk of death during the year post IE (HR 1.41, 95% CI 1.23–1.61). An age ≥70 years (HR 1.90, 95% CI 1.72–2.10), cancer (HR 1.26 95% CI 1.13–1.41), chronic liver disease (1.76, 95% CI 1.54–2.02), *Staphylococcus aureus* infection (HR 1,74 95% CI 1.54–1.97), *Candida* sp. (HR 1.90, 95% CI 0 1.26–2.86), polymicrobial IE (HR 1.32, 95% CI 1.12–1.56), ischemic or hemorrhagic stroke (HR 1.48 and 1.97, respectively) were significantly associated with one-year mortality. Intravenous drug use (HR 0.32, 95% CI 0.19–0.53), obesity (HR 0.83, 95% CI 0.72–0.96), streptococcal/enterococcal IE (HR 0.80, 95% CI 0.70–0.91) were associated with one-year survival.

**Table 3:** Factors associated with initial and one-year mortality.

Variable	Mortality during initial stay adjusted Odds Ratio (95%CI), Multivariate logistic regression	One-year mortalityAdjusted Hazard ratio (95%CI),Cox model*
Age ≥ 70 years	1.98 (1.75–2.25)	1.90 (1.72–2.10)
Predisposing diseases		
Cancer	1.68 (1.45–1.95)	1.26 (1.13–1.41)
Chronic respiratory disease	1.25 (1.07–1.46)	-
Chronic liver disease	2.65 (2.18–3.23)	1.76 (1.54–2.02)
IDU	0.26 (0.15–0.47)	0.32 (0.19–0.53)
Obesity	-	0.83 (0.72–0.96)
Dialysis	1.59 (1.23–2.05)	- -
Microorganism (monomicrobial)		
*Streptococci/Enterococci*	-	0.80 (0.70–0.91)
*Staphylococcus aureus*	2.01 (1.71–2.36)	1.74 (1.54–1.97)
CNS	1.40 (1.09–1.80)	-
Non *E*. *coli* NGB	1.52 (1.03–2.24)	-
*Candida* sp	3.01 (1.62–5.61)	1.90 (1.26–2.86)
Code for non-specified pathogen	1.55 (1.16–2.08)	1.43 (1.15–1.80)
. Polymicrobial	1.69 (1.35–2.10)	1.32 (1.12–1.56)
Cerebral complications		
Ischemic stroke	1.84 (1.52–2.22)	1.48 (1.28–1.70)
Hemorrhagic stroke	2.79 (2.08–3.75)	1.97 (1.62–2.40)
Valvular surgery	0.47 (0.41–0.55)	0.83 (0.71–0.97)

* multivariate Cox modelling with time-dependent covariate.

## Discussion

We report the national epidemiology of IE in France, based on a retrospective population-based cohort study. The estimated national incidence (63 cases/million inhabitants) was higher than previously reported in France [9,15]. Our results are in line with our previous study [14]. This time, we performed a first step of validation in a different cohort, using a sample of medical reports from 7 French hospitals (4 teaching hospitals and 3 general hospitals), which yielded a PPV of 86.4% for the diagnosis of IE according to Duke criteria, and 75.1% for definite IE. This confirms that the definition based on the electronic medico-administrative database is fairly robust, and that the study population is representative of the French population of IE. HD data are usually used for an economical purpose, but their epidemiological use is increasing, notably in the field of infectious diseases [16,17]. The case-definition based on ICD-10 diagnoses has to be sensitive and robust (admitted target PPV >75% for surveillance purpose) [18,19]. Hence, the reliability of national health HD systems for epidemiology may vary significantly according to case definition and could generate false positive diagnosis, whereas false negative rate remained relatively low whatever the definition [18,19]. Selection of IE cases based on hospitalization data appears reasonable given that almost all cases of IE are managed as inpatient, at least initially. The mean hospital stay was 31 days, similar to recent IE literature [20].

The annual incidence, adjusted on PPV and frequency of definite IE, was higher than reported in France by Selton-Suty *et al* (63 vs. 33.8 cases per million inhabitants). Our results based on hospital exhaustive data may represent a snapshot of the real epidemiology of IE in France. The Selton-Suty study was a prospective observational study, which relied on notification of cases by investigators, which may be at higher risk of underreporting as compared to HD, hence potentially responsible for underestimation of IE incidence, as in other European studies [4,6]. Conversely, different algorithms used to detect IE from HDD found either lower [9], or higher estimates of incidence [12,13]. However, the regular control of the HDD in France by the National Health Insurance ensures a fair quality of coding, which was confirmed in one large validation panel in our study. The incidence of IE is increasing with age and predominant in male, as previously described [21]. For example, in people aged from 80 to 89 years, incidence of IE was 500 per million inhabitants in males vs. 250 per million inhabitants in females [2,22]. This higher incidence could be explained by the higher prevalence of valvular diseases and valvular prostheses in elderly and males, along with other comorbidities known to promote bacterial infections, and decreased immunity with ageing [23–26].

The prevalence of valve surgery in our study was lower than reported by Selton-Suty *et al* (25.3% vs 44.9%), but comparable to other studies [11,27]. Some authors suggested that the incidence of cardiac surgery had decreased in recent years [28]. In most studies on cardiac surgery for IE, the analysis design does not include patients with theoretical indication for cardiac surgery, but who were ineligible for medical/organisation purposes. In-hospital mortality was similar to previous studies (20.6%) [2,15,29]. It has been demonstrated that patients with indication for cardiac surgery but who do not undergo surgery, have very high in-hospital mortality, up to 80% [2,15,29]. Thus, low rates of cardiac surgery are unlikely to be explained by non-adherence of the surgeon with recommendations for valvular surgery, otherwise in-hospital mortality would be much higher. Indeed, valvular surgery during initial hospitalization was associated with a lower risk of mortality in our study. It is possible that the discrepancy is in part related to referral bias in previous studies. As previously shown, surgical management varies from one center to another [30]. The tertiary university centers of the Selton-Suty study are strongly implicated in IE care, with close collaboration between infectious disease teams, cardiologists and cardiac surgeons (IE teams), not necessarily representative of the IE management for the global population of patients with IE. However, this study tried to include as many different health care facilities as possible in order to be representative of the French healthcare centers. The high level of expertise and the presence of a comprehensive technical platform should have contributed to easier recourse to surgery, even for patients not having formal indications for such intervention. Recent population-based studies found similar rates of valvular surgery with no excess of mortality [6,8,31].

The factors associated with cardiac surgery were similar to previous reports[1,32]. Hence, during the initial hospitalization, younger patients and males were more likely to undergo surgery whereas patients with hemorrhagic stroke, *S*. *aureus*, or *Streptococci/Enterococci* IE were less likely to go to surgery. However, looking at cardiac surgery within the first year after IE diagnosis, patients with streptococcal IE were more likely to undergo surgery whereas patients with *S*. *aureus* IE were less likely to undergo surgery. This could be explained by the severity of IE caused by the *S*. *aureus*, as suggested by our results (higher mortality rates) consistently with the literature [8,33–35]. Intravenous drug users and obesity were associated with survival, maybe because these factors are more associated with right-sided IE (not identificable in our HD study). Valvular surgery during the initial admission was associated with better survival after adjusting on confounders (HR 0.47, CI 95% 0.41–0.55), whereas the surgery during the follow-up was associated with a 1.4-fold higher risk of death within the year (HR 1.4, CI 95% 1.23–1.61).

This study had limitations, especially due to the use of HD. Strengths and limitations of using healthcare databases for epidemiological purposes have already been extensively discussed [14,17,18]. One has to keep in mind that observed changes in disease patterns could be biased by variations in coding practices due to financial incentives to obtain higher reimbursement rates [36]. However, the two steps of validation allowed to minimize these biases linked with retrospective hospital data. This HD-based national cohort study is an innovative idea, allowing the identification of rare events, such as IE. Further investigations are needed to better evaluate whether clinical data extracted from health administrative information systems could improve IE knowledge and surveillance, including to analyze the potential impact of surgery. Validation of health information system algorithms through data collected from inpatient medical reports is a necessity. If the advantage of the French HD database is its nationwide coverage offering a global picture of hospital healthcare pathways, its first purpose remains economical and the data collected are not always the more accurate for a given study. For instance, our results concerning microorganisms responsible for IE could be biased by the coding process. Lastly, surgical procedure dates are monthly reported in the hospital discharge database (MM/YYYY), which reduces the precision of time delay estimation between IE and surgery. However, using hospital lengths of stay, survival curves and cox analyses have been performed. To better assess the use of health information systems in IE, efforts remain to be done on the data quality, the detection and decision framework. Nevertheless such health administrative information systems offer real opportunities for public health research on care trajectories. Finally, despite some limitations, HDD-based surveillance should be promoted as a cost-effective method for IE surveillance studies, and could be an alternative to usual surveillance systems, especially for low-prevalence diseases. Cost-benefit analysis and studies combining multiple hospital databases are then warranted.

This national study, following a regional preliminary study [14], demonstrates the feasibility of using health insurance administrative information system to study national IE care trajectories allowing patient follow-up and identification of factors associated with mortality. It could represent a potential tool for IE monitoring and foster the automation of surveillance. The study presented herein suggests that IE incidence in France may be much higher than previously reported.

## Supporting information

S1 Appendix Algorithm of infective endocarditis selection. (DOCX)
