# Supplementary material for: Incidence, characteristics, and mortality of infective endocarditis in France in 2011
Source: PLoS One. 2019 Oct 25;14(10):e0223857. doi: 10.1371/journal.pone.0223857 (PMC6814232; doi:10.1371/journal.pone.0223857)
Supplement: S1 Appendix — (DOCX) [file pone.0223857.s001.docx]

**S1 Appendix – Algorithm of infective endocarditis selection**

The patients were selected on the basis of an HD algorithm developed by experts specializing in infectious diseases and in medical information systems. Cardiologists agreed this HD algorithm. Each stay with a principal or secondary diagnosis code (International Classification for Diseases – ICD 10) of IE appearing alone or in combination with either microbial or associated complication codes (Table).

ICD-10 code associations for case definition:

| Association of codes | **Primary diagnosis** | **Secondary diagnosis** |
| --- | --- | --- |
| 1 | EI |  |
| 2 | Complication | EI |
| 3 | Infection | EI |
| 4 |  | EI **and** infection |
| 5 | Valvular prosthesis infection |  |
| 6 |  | EI **and** microorganism |
| 7 | Bacteriemia | Valvular prosthesis infection |

ICD-10 codes for the infective endocarditis (IE) selection

Bacteriemia codes: A40 A78 B95 A410 A411 A412 A419 A440 A448 A490 A491 A499 B376 B377

EI codes: I33 I38 I39

Valvular prosthesis infections codes: T826

Infection codes: A40 A48 A78 B95 A395 A410 A411 A412 A413 A414 A415 A418 A419 A448 A449 A478 A479 B376 B377

Microorganism codes: A490 A491 A493 A498 A499 B960 B961 B962 B963 B964 B965 B966 B968

Complication codes: I340 I348 I349 I351 I352 I358 I359 I361 I362 I368 I369 I371 I372 I378 I379 I440 I441 I442 I443 I459 R570 I501 I509 I500 I63 I64 I74 G459 G458 D735 N280 I260 I269 I60 I61 I724 I723 I729 M00 G003 G060 G061 M016 M461 M462 M463 M464 M465 M860 M861 M869 J853 R572 M036 N088
